# X-ray fluorescence analysis of metal distributions in cryogenic biological samples using large-acceptance-angle SDD detection and continuous scanning at the Hard X-ray Micro/Nano-Probe beamline P06 at PETRA III

**DOI:** 10.1107/S1600577519014048

**Published:** 2020-01-01

**Authors:** C. Rumancev, A. Gräfenstein, T. Vöpel, S. Stuhr, A. R. von Gundlach, T. Senkbeil, J. Garrevoet, L. Jolmes, B. König, G. Falkenberg, S. Ebbinghaus, W. H. Schroeder, A. Rosenhahn

**Affiliations:** a Analytical Chemistry – Biointerfaces, Ruhr University Bochum, 44780 Bochum, Germany; b Department of Physical Chemistry II, Ruhr University Bochum, 44780 Bochum, Germany; c Deutsches Elektronen-Synchrotron DESY, Notkestrasse 85, Hamburg, Germany; d Institute of Physical and Theoretical Chemistry, TU Braunschweig, Rebenring 56, 38106 Braunschweig, Germany; e Nanotech Consulting, Arnoldsweilerstrasse 10, 52382 Niederzier, Germany

**Keywords:** solid angle, SDD, XRF, cryogenic, HeLa cells

## Abstract

A new Rococo 2 X-ray fluorescence detector was implemented into the cryogenic sample environment at the Hard X-ray Micro/Nano-Probe beamline P06 at PETRA III, DESY, Hamburg, Germany. A four sensor-field cloverleaf design is optimized for the investigation of planar samples and operates in a backscattering geometry resulting in a large solid angle of up to 1.1 steradian. The detector, coupled with the Xspress 3 pulse processor, enables measurements at high count rates of up to 10^6^ counts per second per sensor. The measured energy resolution of ∼129 eV (Mn *K*α at 10000 counts s^−1^) is only minimally impaired at the highest count rates. The resulting high detection sensitivity allows for an accurate determination of trace element distributions such as in thin frozen hydrated biological specimens. First proof-of-principle measurements using continuous-movement 2D scans of frozen hydrated HeLa cells as a model system are reported to demonstrate the potential of the new detection system.

## Abstract 2

A new Rococo 2 X-ray fluorescence detector was implemented into the cryogenic sample environment at the Hard X-ray Micro/Nano-Probe beamline P06 at PETRA III, DESY, Hamburg, Germany. It features a high solid angle of up to 1.10 steradian and high count rates of 1 Mcounts s^−1^ per sensor.

## 1. Introduction   

Nano- and microprobe X-ray fluorescence (XRF) analysis at synchrotron sources provides access to map and visualize the distribution of chemical elements in biological samples and is thus a technique commonly used in biology and medicine (Paunesku *et al.*, 2006 ▸; Börjesson *et al.*, 2003 ▸). It was applied for *in vitro* measurements of lead in bones (Todd & Chettle, 1994 ▸) and to detect trace element distribution in plants (Punshon *et al.*, 2009 ▸). Among recent biomedical applications of XRF are the determination of the elemental content in human neutrophils (De Samber *et al.*, 2016 ▸; Niemiec *et al.*, 2015 ▸), in mouse embryonic fibroblast cells (Matsuyama *et al.*, 2010 ▸), in PC12 cells (Kosior *et al.*, 2012 ▸), in Alzheimer-affected brain cells (Miller *et al.*, 2006 ▸), melanosomes (Gorniak *et al.*, 2014 ▸) and dopamine neurovesicles (Miller *et al.*, 2006 ▸). In environmental applications, XRF was used to analyze single bacterial cells (Kemner *et al.*, 2004 ▸), juvenile barnacles (Senkbeil *et al.*, 2016 ▸) and damselfly wings (Stuhr *et al.*, 2018 ▸). Recent trends show the potential to push the method towards 3D XRF tomography (De Samber *et al.*, 2010*a* ▸,*b* ▸; Antipova *et al.*, 2018 ▸; de Jonge & Vogt, 2010 ▸; Takeuchi *et al.*, 2009 ▸; Martínez-Criado *et al.*, 2016 ▸). In particular for tomography, a rapid acquisition of the large amount of data is desired to reduce drift effects. In recent years, significant technological improvements have been made including advanced X-ray sources, improved focusing schemes (Seiboth *et al.*, 2017 ▸) and better detectors (West *et al.*, 2012 ▸; Wu *et al.*, 2014 ▸). Besides the focused X-ray beam, the energy-dispersive detector in particular for analyzing the emitted X-rays is of key relevance for every analysis system. Silicon drift detectors (SDDs) are commonly used for energy-resolved detection of ionizing radiation like X-rays. The first SDD was developed by Gatti & Rehak (1984 ▸). The function principle is similar to a photodiode but field strips drive the signal charges towards a small collecting anode with very low capacitance (Lechner *et al.*, 1996 ▸). The advantages of this detection scheme are the high detection efficiencies due to the rapid counting of all collected photons with different energies while still providing a good energy resolution. Another advantage is that SDDs work at moderately low temperatures which allows the use of Peltier cooling instead of liquid-nitro­gen cooling. Currently, different beamlines at synchrotron sources are aiming to maximize the detection efficiency of the detectors (Gianoncelli *et al.*, 2015 ▸; Lühl *et al.*, 2019 ▸). For instance, the development of the Maia detector array has been a milestone for high-throughput fluorescence analysis, using an annular, back-scattering geometry with a 20 × 20 array of 1 mm^2^ individual detectors, capturing a high solid angle at high count rates (Ryan *et al.*, 2019 ▸).

In this work we use the Rococo 2 SDD (PNDetector, München, Germany) which was developed for energy-dispersive X-ray spectroscopy (EDX) (Schlosser *et al.*, 2010 ▸) in electron microscopy. It uses four separated detection elements that cover a large acceptance angle of up to 1.1 steradian and is equipped with a 1 µm Mylar window and a zirconium mask. We integrated the Rococo 2 detector into the cryogenic sample environment at the microprobe experiment of the Hard X-ray Micro/Nano-Probe beamline P06 and tested it for the first time at a synchrotron source in a vacuum environment using cryogenically prepared samples. Cryogenic sample environments are a powerful tool for investigating rapidly frozen hydrated biological specimens, which are as close as possible to their natural aqueous environment (Dubochet, 2012 ▸). In addition, the visible effects of radiation damage are strongly reduced (Schneider *et al.*, 1995 ▸). As the experiments were carried out at the microprobe branch of the high-brilliance P06 beamline of the synchrotron PETRA III, ultrafast pulse processors were required to analyze the emitted XRF photons. The high count rates allow a short acquisition time, which is crucial to effectively exploit the beam time at synchrotron sources. A high sensitivity and good energy resolution are also mandatory to detect trace elements, and the ultrathin window improves the detection efficiency of light elements like potassium, sulfur or phospho­rus by reducing the absorption of the corresponding signal to a minimum. The latter are elements of importance in many biological processes. Due to the high penetration depth of hard X-rays, XRF allows relatively thick samples such as cells or tissue sections to be quantitatively analyzed. Here we characterize the Rococo 2 detector regarding its energy resolution, maximum count rates and dead-time, and show first XRF measurements of cryogenically preserved HeLa cells with this new detector concept.

## 2. Material and methods   

### 2.1. Sample preparation   

HeLa cells were cultured in DMEM (Dulbecco’s Modified Eagle’s Medium; Gibco, Carlsbad, USA) supplemented with 10% fetal bovine serum (Sigma Aldrich Chemie GmbH, Steinheim am Albuch, Germany) and 1% penicillin-streptomycin (Gibco, Carlsbad, USA) at 37°C and humidified atmosphere with 5% CO_2_ using T-25 cell culture flasks (Sarstedt AG & Co, Nümbrecht, Germany). 7500–10000 cells were split and seeded onto silicon nitride membranes (1 mm × 1 mm; Silson Ltd, Blisworth, UK). Cells were incubated in culture medium for another 12–16 h prior to imaging with a light microscope. During the light microscopy the cells were placed in a phosphate-buffered saline (PBS) (catalogue number 18912-014; Gibco, Inchinnan, UK) solution. Subsequently, cells on their support membranes were plunge-frozen in a liquid-ethane/propane mixture and stored in liquid nitro­gen until the XRF measurements.

### 2.2. X-ray fluorescence measurement and data analysis   

**Figure 1:**
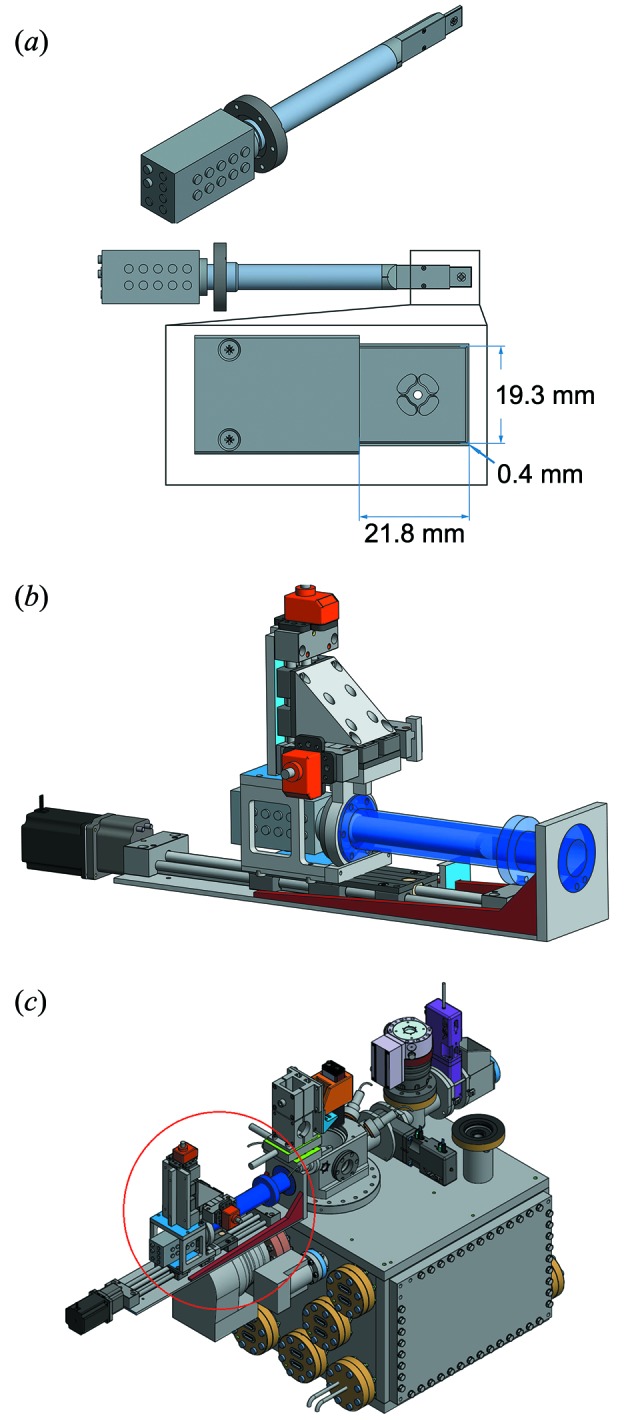
(*a*) CAD drawings of the Rococo 2 detector. The active area (4 × 15 mm^2^) of the detector is depicted in the inset. The thickness of the active elements is 450 µm. It is equipped with a 1 µm Mylar window and a zirconium mask (21.8 mm × 19.3 mm × 0.4 mm) and optimized for a 2 mm sample distance (solid angle = 1.1 steradian), which corresponds to a working distance of ∼6 mm (courtesy of PNDetector, Munich, Germany). (*b*) Mounted Rococo 2 detector on the *XYZ*-manipulator. The manipulator can be directly attached to the vacuum chamber. The structure in blue is a flexible vacuum bellow. (*c*) Cryogenic vacuum chamber at the P06 beamline with the integrated Rococo 2 detector on the *XYZ*-manipulator (red circle).

Micro-XRF measurements were carried out at the microprobe branch of the Hard X-ray Micro/Nano-Probe beamline P06, DESY (Hamburg, Germany) (Schroer *et al.*, 2010 ▸). A cryogenic vacuum chamber with a modified recipient for the detector and for the sample transfer system was used. It enables measurements in vacuum at a base pressure between 10^−6^ mbar and 10^−8^ mbar at a typical sample temperature of 120 K. The synchrotron beam (12 keV) was focused to a size of 300 nm × 300 nm. The entrance window into the cryogenic vacuum chamber was made of black Kapton with a thickness of 75 µm (Dupont, Hertfordshire, UK). The fluorescence signal was detected by the Rococo 2 detector, which uses four monolithically integrated SDD sensor elements (15 mm^2^ active area each) in an annular cloverleaf shape centered around a hole with a 1.8 mm diameter to allow transmission of the synchrotron beam [Fig. 1(*a*) ▸]. This enables the use of the detector in close proximity to flat samples at a high acceptance angle of up to 1.1 steradian. The detector was positioned in a backscatter geometry, 3 mm upstream of the sample, corresponding to a working distance of ∼7 mm (solid angle ≃ 1.0 steradian). The 2D area scans were measured in continuous-scan mode, in which the sample stages were continuously moved in the horizontal direction, and at each scan end moved incrementally in the vertical direction. During measurements the spectral data were collected for a pre-defined time over a pre-defined distance using an Xspress 3 pulse processor (Quantum Detectors, Oxford) – featuring the high processing speed necessary for the Rococo 2. The precise position of the stages was measured using encoders. The intensity of the excitation X-ray beam was monitored using an ionization chamber before and a photodiode behind the cryogenic chamber. For the calibration and characterization measurements, the spectra of the four active elements of the Rococo 2 detector were summed up and normalized to the intensity of the excitation beam. For the 2D scans of the cryogenically prepared cells only three of the four elements were summed up, as one of the readout channels was used for diagnostic purposes. In continuous scan mode, the spectra were assigned to pixel positions on a Cartesian grid according to the scan parameters using the recorded encoder positions. All spectra were analyzed by peak deconvolution and fitting using the fast XRF stacking function of the software *PyMCA* (European Synchrotron Radiation Facility, ESRF) (Solé *et al.*, 2007 ▸). The thickness of the amorphous ice of the rapid plunge frozen HeLa cells was determined by comparing the intensity of the mean Si *K* signal *I*(*K*_Si,ice_) at the position of the cells with the Si *K* signal obtained from a spectrum of an empty silicon nitride membrane [*I*(*K*_Si,membrane_)]. By equating the ratio *I*(*K*_Si,ice_):*I*(*K*_Si,membrane_) to the transmission of the Si *K* signal at 1.74 keV (attenuation length in water λ = 11.6 µm) (Henke *et al.*, 1993 ▸) and using Lambert–Beer’s law, the thickness of the amorphous ice (ρ = 0.94 g cm^−3^) can be determined via the following equation,
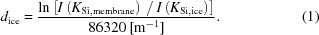



## 3. Results   

The Rococo 2 detector was implemented in the cryogenic vacuum chamber to improve the sensitivity for XRF microscopy. Fig. 1(*a*) ▸ shows a CAD drawing of the detector. The detector was mounted on a custom-built *XYZ*-manipulator [Fig. 1(*b*) ▸], which allows alignment to the beam through the detector aperture and to adjust the detector–sample distance to ∼3 mm. In conjunction with the KB focus of the beamline, 2D scans can be rapidly acquired, which is particularly relevant for analysis of trace elements in biological samples. The motorized setup enables a quick alignment and to return to the short sample–detector distances for a large acceptance angle after sample transfer.

### 3.1. Energy resolution   

**Figure 2:**
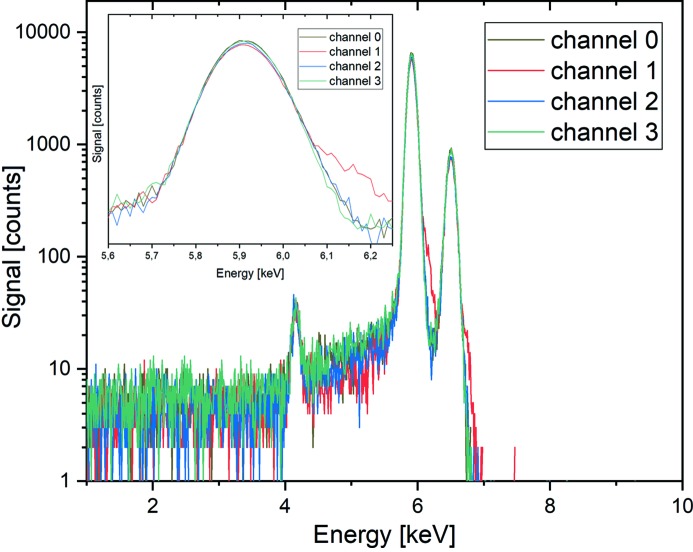
Energy resolution test of the individual four active elements of the detector using a ^55^Fe radioactive source emitting the Mn *K* characteristic lines at 5.9 keV and 6.5 keV. The inset shows that the *K*α signal of the four segments is very similar.

Before its use at the beamline, the energy resolution of the detector was characterized in a laboratory setup at ∼22°C (with the detector being cooled to −25°C) in a high-vacuum chamber (10^−6^ mbar) using a ^55^Fe radioactive source which emits the Mn *K* characteristic lines. The signal detected by the four active elements of the Rococo 2 are shown in Fig. 2 ▸ (see also Table 1 ▸). The signals of all four detector channels are similar regarding their shape. Gaussian fits of the Mn *K*α line for each of the channels yielded a mean full width at half-maximum (FWHM) of 128.7 ± 0.9 eV @ 10 kcounts s^−1^, which is in good agreement with the specifications of the manufacturer of a FWHM of the Mn peak of 128 eV @ 100 kcounts s^−1^ (Rococo Preamplifier Module, https://pndetector.com/w/wp-content/uploads/2018/08/Rococo_2017.pdf). A peak-to-background ratio of approximately 1300:1 was determined.

### 3.2. Count rate and dead-time   

**Figure 3:**
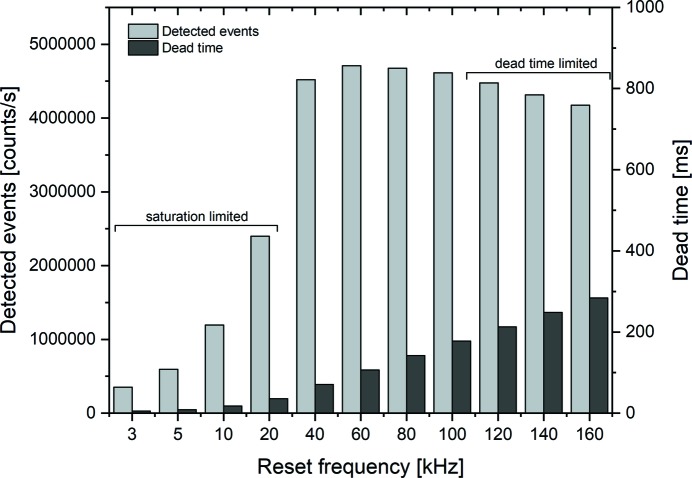
Left axis: detected events by the Rococo 2 detector summed over all four elements over the entire spectrum at different fixed reset frequencies. The acquisition time was 1 s. Right axis: total reset pulse dead-time for the corresponding frequencies. The frequency areas in which either saturation or the pulse dead-time was the major limiting factor for the count rate are marked.

Like in any SDD detector, the accumulating charge has to be periodically reset in order to prevent saturation of the anode. The current version of the detector was operated with a constant reset frequency that could be modified by the operator. At high count rates, typical at synchrotron sources, the required high reset rate causes a significant dead-time that may limit the obtained count rate. In order to evaluate the optimal reset time of the detector/pulse processor system, we varied the reset frequencies from 3 kHz to 160 kHz and determined the effective count rate. Fig. 3 ▸ shows the sum of all detected counts during the acquisition time of 1 s summed across all four channels. The dark bars represent the counts recognized by the pulse processor with automatically subtracted background, while pile-up peaks were still considered in the analysis. The obtainable count rate increases towards higher reset frequencies about tenfold from 3 kHz to the maximum at 60 kHz, where a count rate of 4.7 × 10^6^ counts s^−1^ was reached. At this reset frequency, a dead-time of 107 ms (11%) can be estimated. For frequencies lower than 60 kHz, the lower count rate is due to the saturation of the respective anodes of the active elements before reset. For frequencies above 60 kHz, the count rate again decreases due to the dead-time caused by the reset, each one taking 1.775 µs, which means that the total reset pulse dead-time per second increases from 5.33 ms at 3 kHz (5%) to 107 ms at 60 kHz (11%) to 284 ms at 160 kHz (28%). Additionally, an increase of the reset frequency leads to a decrease of the voltage ramp step length on the time axis. If the step length is shorter than the process time *T*_p_, the photon event will not be counted. Hence, at increasing reset frequencies, the proportion of rejected events will increase as well, leading to an overall decrease of counted events. Also, the reduction of the step length leads to worse averaging of the voltage noise, which in return can lead to a broadening as well as energy shifts of the measured elemental signal peaks. To a small extent, this was already visible in the spectra we measured at the reset frequency of 60 kHz. In particular, we noticed a general energy shift of the elemental peaks of ∼10% as well as a broadening of the higher energy side of the peak. The shift was easily accounted for using a linear correction. A new version of the XRF Rococo 2 detector with automated peak-reset unit is currently in development and will soon be integrated.

### 3.3. 2D Scans of cryogenically analyzed HeLa cells attached to a silicon nitride membrane   

**Figure 4:**
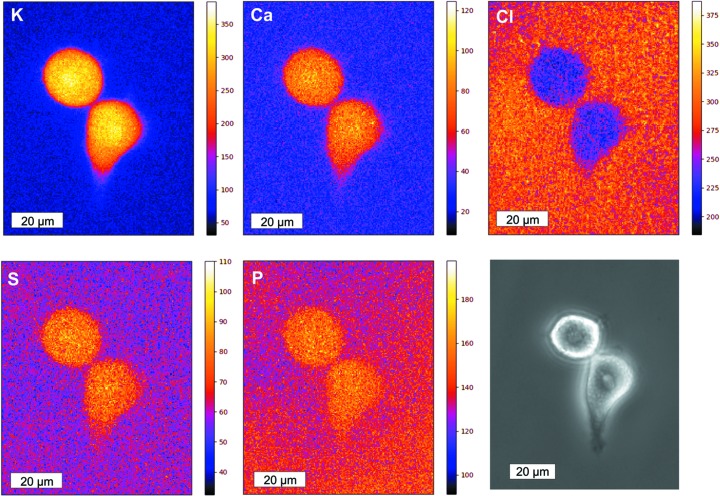
XRF single element maps (180 × 221 pixels per map) obtained from HeLa cells with corresponding light microscopic image. The scale bar for the XRF maps and the light microscopy image is 20 µm. The color bar to the right of the XRF maps represents the total integrated counts of the detector per pixel during the selected acquisition time. The XRF maps were recorded with a 400 nm pixel size and an exposure time of 0.03 s. The total scanning duration was 30 min.

For a further characterization of the detector for trace metal analysis and to demonstrate the capabilities of the setup, HeLa cells prepared by rapid plunge-freezing were investigated in a frozen-hydrated state. 2D XRF scans of frozen hydrated HeLa cells were obtained in continuous-scanning mode, but only three out of the four channels were used for detection as the fourth one was used for diagnostic purposes during the scanning process. Two-dimensional maps were created in order to reveal the distribution of the detected elements (see Section 2.2). As exposure time per pixel, a value between 0.03 and 0.1 s was typically sufficient to obtain a sufficient count rate. This led to total scanning times of ∼10 min for a 50 µm × 50 µm scan with a 400 nm pixel size and an exposure time of 0.03 s per pixel. In Fig. 4 ▸, XRF maps of selected elements of physiological interest (potassium, calcium, chlorine, phospho­rus and sulfur) of typical HeLa cells are shown. The scanned area is 88 µm × 72 µm with an exposure time of 0.03 s and a pixel size of 400 nm. The total acquisition time was 30 min for the shown scan. The corresponding line profiles across the HeLa cells are provided in Fig. S1 of the supporting information. All elements shown in Fig. 4 ▸ except for chlorine show an elevated signal inside the two HeLa cells compared with the extracellular surrounding, with the map of potassium displaying the highest dynamic range of 60:1. The obvious steep gradient of potassium across the cell is a strong indication that the cell membranes are still intact. The signal intensity in the element maps is roughly uniformly distributed inside the cells with a lower intensity towards the cellular periphery. This was expected since the thickness of the cells decreases towards the edges. The concentration of chlorine, on the other hand, appears to be lower inside the cells compared with the surrounding area. The chloride concentration in the PBS medium used during shock freezing is known to be 143 mM. Under the assumption that the chloride concentration gradient across the cell membrane must be nearly the same (in the opposite direction) as the K^+^ concentration gradient, and under the likely assumption that the resting potential across the membrane is undisturbed (supported by the shown images, especially K), the chloride concentration inside the cells [according to Hodgkin & Horowicz (1959 ▸)] should be approximately 7.5 mM, which is much lower than that in the surrounding medium. The lower intensity of the Cl signal within the cells is thus caused by the volume occupied by the cell with a lower chloride concentration. Using the method described in Section 2.2, we could determine an ice thickness of 17.4 µm at the position of the cells (compared with a typical cell height of 8–10 µm), while outside the cells an ice thickness of 16.5 µm could be determined. In general, the variance of the ice thickness throughout the scan was very low and local differences were below 1 µm. Since the ratio of the chloride signal intensity inside the cell to the chloride signal intensity outside the cells in the Cl map in Fig. 4 ▸ is smaller than expected from the literature concentrations mentioned above (1.22 versus 19), one can conclude that the cells must be completely covered with ice with the chloride signal originating mostly from the intact and closed ice layer above the cells.

**Figure 5:**
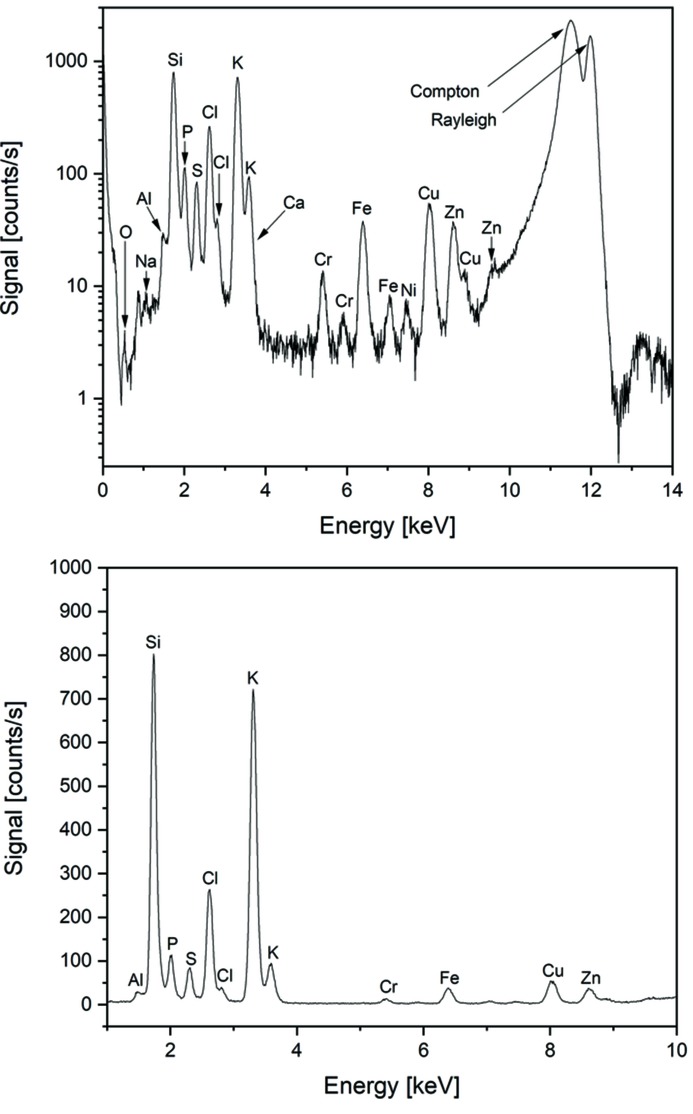
XRF sum spectrum over 100 pixels obtained with the Rococo 2 detector on a single HeLa cell, in log scale (top) and in linear scale (bottom). Total exposure time was 10 s. The spectrum was collected in a focus size of 300 nm × 300 nm of the X-ray beam using an excitation energy of 12 keV.

In Fig. 5 ▸, a sum spectrum obtained over 100 pixels in the middle of another HeLa cell is shown on a log scale (top) and on a linear scale (bottom). The incoming photon flux was 5.7 × 10^9^ photons s^−1^ and the total acquisition time for the spectrum was 10 s (100 × 0.1 s). Typical relevant biological elements like phospho­rus, sulfur, chlorine and potassium are visible in the spectrum. The fluorescence energy of the light elements aluminium, sodium and even oxygen can be clearly resolved in the spectrum. Thus, a minimal lower energy threshold of 0.5 keV can be estimated for the Rococo 2 detector under optimal conditions. For the elements Cl, K, Cr, Fe, Cu and Zn, the *K*β signal can be seen in the logarithmic plot in addition to the *K*α signal. The calcium signal is overlapped by the more pronounced potassium *K*β signal. The silicon signal in part arises from the silicon nitride membrane which was used as sample support for the HeLa cells. In addition, the transition metals iron and copper are clearly visible in the spectra of the Rococo 2 detector but are homogeneously distributed throughout the scans without any correlation to the cell morphology in the XRF maps. The metal signals could be caused by X-rays scattered from the amorphous ice that interact with metal parts in the sample holder, by impurities in the sample holder or deposits from cell culture. As the sample holder is made of copper, the excitation of XRF of these metal parts by X-rays scattered at imperfections in the vitreous ice matrix is the most probable explanation.

Peak heights of 722 counts s^−1^ and 803 counts s^−1^ could be determined for the K *K*_α_-peak and for the Si *K*_α_-peak. The peak-to-background ratio of the peaks, which was derived using an averaged background from 4 keV to 5 keV in the spectrum, is 241:1 for K and 269:1 for Si, and hence is lower than the ratio 1300:1 obtained with the ^55^Fe radiative source. The lower peak-to-background values as compared with the benchmark measurements above are most likely due to the attenuation of the XRF signals in the ice matrix, which is significant in particular for the light elements. In addition, the background in the HeLa cell spectrum is elevated in the regime from 4 keV to 5 keV due to the contribution of the Compton scattering. The discussed properties derived from the spectrum in Fig. 5 ▸ are summarized in Table 2 ▸.

In the case of the presented data, only three of the four readout channels were used for data acquisition as the fourth one was used for diagnostic purposes. A further detector signal increase by a factor of 1.33 is expected with the full active area of 60 mm^2^ for all shown and discussed HeLa cell spectra.

In order to estimate the detection limit of the Rococo 2 detector for trace element analysis, an XRF spectrum on a thin film XRF reference sample (AXO Dresden, Germany) was obtained, from which a trace detection limit between 50 and 95 parts per million could be estimated (see Fig. S3 of the supporting information).

## 4. Discussion and conclusion   

A new Rococo 2 X-ray fluorescence detector was introduced at the P06 beamline at DESY. The Rococo 2 is equipped with an active area of 60 mm^2^ and enables a solid angle up to 1.1 steradian. Combined with high count rates and energy resolution the Rococo 2 provides high-quality measurements on biological, cryogenically prepared samples in a high-throughput process. In the first laboratory test a mean energy resolution of 128.7 ± 0.9 eV and a peak-to-background ratio of ∼1300:1 could be measured over all four readout channels using a ^55^Fe radioactive source. A maximum count rate of 4.7 × 10^6^ counts s^−1^ could be achieved at a reset dead-time of 107 ms (11%). As biological test system for trace element analysis, rapid plunge-frozen HeLa cells were imaged under cryogenic conditions using the new Rococo 2 detector. Here we used the detected signals of two different elements, silicon and potassium, to estimate the properties of the Rococo 2 detector. We detected a peak-to-background ratio of 241:1 for potassium and 269:1 for silicon with a FWHM of the potassium peak of 113 eV.

Particularly when compared with the recent setup at our chamber using a conventional SDD module with an active area of 50 mm^2^ and a relatively low acceptance angle of 0.066 steradian (due to a larger sample-to-detector distance), the new detector offers a much larger acceptance angle of 1.0 steradian at a *z* distance of 3 mm, leading to a reduction of measurement time by a factor of ∼30. The additional benefits of the new implementations are the large dynamic range and a high signal-to-noise ratio. In Fig. S2 of the supporting information a comparison between the XRF spectrum in Fig. 5 ▸ and an XRF spectrum of a similar HeLa cell acquired with the old detector setup is shown.

The Rococo 2 detector with the ability to deliver high count rates and its high sensitivity and energy resolution enables intracellular metal analysis and the scanning of large areas of tissue or cells in a high-throughput approach with minimal scanning durations. Particularly for the analysis of samples which need to be kept in a native state but chemical fixation and staining are not an option, the improved cryogenic environment with the Rococo 2 detector at the microprobe branch of the P06 beamline under vacuum provides a unique analysis opportunity for researchers from biology.

## Supplementary Material

Figure S1: intensity line profiles. Fig. S2: Comparision of XRF spectra for the new and old detector setups. Fig. S3: XRF spectrum of a reference sample. DOI: 10.1107/S1600577519014048/ok5004sup1.pdf

## Figures and Tables

**Table 1:** Corresponding FWHM values derived from Gaussian fits of the Mn *K*α signal in Fig. 2 ▸

Channel	FWHM (eV)
Channel 0	128.5 ± 0.8
Channel 1	129.1 ± 0.9
Channel 2	128.8 ± 1.0
Channel 3	128.5 ± 0.8
Mean	128.7 ± 0.9

**Table 2:** Signal properties of the Rococo 2 detector as determined from the spectrum in Fig. 5 ▸

	HeLa cell (Fig. 5 ▸)
Peak height, K (counts s^−1^)	722
Peak height, Si (counts s^−1^)	803
Background (counts s^−1^)	2.99
Peak-to-background ratio, K peak	241
Peak-to-background ratio, Si peak	269
FWHM K peak (eV)	113

## References

[bb1] Antipova, O., Kemner, K., Roehrig, C., Vogt, S., Li, L. X. & Gursoy, D. (2018). *Microsc. Microanal.* **24**, 520–521.

[bb2] Börjesson, J., Isaksson, M. & Mattsson, S. (2003). *Acta Diabetol.* **40**, s39–s44. 10.1007/s00592-003-0024-z 14618431

[bb7] De Samber, B., Niemiec, M. J., Laforce, B., Garrevoet, J., Vergucht, E., De Rycke, R., Cloetens, P., Urban, C. F. & Vincze, L. (2016). *PLoS One*, **11**, e0165604. 10.1371/journal.pone.0165604 PMC5094720 27812122

[bb5] De Samber, B., Silversmit, G., De Schamphelaere, K., Evens, R., Schoonjans, T., Vekemans, B., Janssen, C., Masschaele, B., Van Hoorebeke, L., Szalóki, I., Vanhaecke, F., Rickers, K., Falkenberg, G. & Vincze, L. (2010). *J. Anal. At. Spectrom.* **25**, 544–553.

[bb6] De Samber, B., Vanblaere, S., Evens, R., De Schamphelaere, K., Wellenreuther, G., Ridoutt, F., Silversmit, G., Schoonjans, T., Vekemans, B., Masschaele, B., Van Hoorebeke, L., Rickers, K., Falkenberg, G., Szaloki, I., Janssen, C. & Vincze, L. (2010). *Powder Diffr.* **25**, 169–174.

[bb8] Dubochet, J. (2012). *J. Microsc.* **245**, 221–224. 10.1111/j.1365-2818.2011.03569.x 22457877

[bb9] Gatti, E. & Rehak, P. (1984). *Nucl. Instrum. Methods Phys. Res.* **225**, 608–614.

[bb10] Gianoncelli, A., Vaccari, L., Kourousias, G., Cassese, D., Bedolla, D. E., Kenig, S., Storici, P., Lazzarino, M. & Kiskinova, M. (2015). *Sci. Rep.* **5**, 10250. 10.1038/srep10250 PMC4431353 25974639

[bb11] Gorniak, T., Haraszti, T., Suhonen, H., Yang, Y., Hedberg-Buenz, A., Koehn, D., Heine, R., Grunze, M., Rosenhahn, A. & Anderson, M. G. (2014). *Pigm. Cell. Melanoma Res.* **27**, 831–834. 10.1111/pcmr.12278 PMC4150745 24903463

[bb12] Henke, B. L., Gullikson, E. M. & Davis, J. C. (1993). *At. Data Nucl. Data Tables*, **54**, 181–342.

[bb13] Hodgkin, A. L. & Horowicz, P. (1959). *J. Physiol.* **148**, 127–160. 10.1113/jphysiol.1959.sp006278 PMC1363113 14402240

[bb4] Jonge, M. D. de & Vogt, S. (2010). *Curr. Opin. Struct. Biol.* **20**, 606–614. 10.1016/j.sbi.2010.09.002 20934872

[bb14] Kemner, K. M., Kelly, S. D., Lai, B., Maser, J., O’loughlin, E. J., Sholto-Douglas, D., Cai, Z., Schneegurt, M. A., Kulpa, C. F. & Nealson, K. H. (2004). *Science*, **306**, 686–687. 10.1126/science.1103524 15499017

[bb15] Kosior, E., Bohic, S., Suhonen, H., Ortega, R., Devès, G., Carmona, A., Marchi, F., Guillet, J. F. & Cloetens, P. (2012). *J. Struct. Biol.* **177**, 239–247. 10.1016/j.jsb.2011.12.005 22182730

[bb16] Lechner, P., Eckbauer, S., Hartmann, R., Krisch, S., Hauff, D., Richter, R., Soltau, H., Strüder, L., Fiorini, C., Gatti, E., Longoni, A. & Sampietro, M. (1996). *Nucl. Instrum. Methods Phys. Res. A*, **377**, 346–351.

[bb17] Lühl, L., Andrianov, K., Dierks, H., Haidl, A., Dehlinger, A., Heine, M., Heeren, J., Nisius, T., Wilhein, T. & Kanngießer, B. (2019). *J. Synchrotron Rad.* **26**, 430–438. 10.1107/S1600577518016879 30855252

[bb18] Martínez-Criado, G., Villanova, J., Tucoulou, R., Salomon, D., Suuronen, J.-P., Labouré, S., Guilloud, C., Valls, V., Barrett, R., Gagliardini, E., Dabin, Y., Baker, R., Bohic, S., Cohen, C. & Morse, J. (2016). *J. Synchrotron Rad.* **23**, 344–352. 10.1107/S1600577515019839 PMC5297598 26698084

[bb19] Matsuyama, S., Shimura, M., Fujii, M., Maeshima, K., Yumoto, H., Mimura, H., Sano, Y., Yabashi, M., Nishino, Y., Tamasaku, K., Ishizaka, Y., Ishikawa, T. & Yamauchi, K. (2010). *X-ray Spectrom.* **39**, 260–266.

[bb20] Miller, L. M., Wang, Q., Telivala, T. P., Smith, R. J., Lanzirotti, A. & Miklossy, J. (2006). *J. Struct. Biol.* **155**, 30–37. 10.1016/j.jsb.2005.09.004 16325427

[bb21] Niemiec, M. J., De Samber, B., Garrevoet, J., Vergucht, E., Vekemans, B., De Rycke, R., Björn, E., Sandblad, L., Wellenreuther, G., Falkenberg, G., Cloetens, P., Vincze, L. & Urban, C. F. (2015). *Metallomics*, **7**, 996–1010. 10.1039/c4mt00346b 25832493

[bb23] Paunesku, T., Vogt, S., Maser, J., Lai, B. & Woloschak, G. (2006). *J. Cell. Biochem.* **99**, 1489–1502. 10.1002/jcb.21047 17006954

[bb25] Punshon, T., Guerinot, M. L. & Lanzirotti, A. (2009). *Ann. Bot.* **103**, 665–672. 10.1093/aob/mcn264 PMC2707871 19182222

[bb26] Ryan, C. G., Siddons, D. P., Kirkham, R., Li, Z. Y., de Jonge, M. D., Paterson, D. J., Kuczewski, A., Howard, D. L., Dunn, P. A., Falkenberg, G., Boesenberg, U., De Geronimo, G., Fisher, L. A., Halfpenny, A., Lintern, M. J., Lombi, E., Dyl, K. A., Jensen, M., Moorhead, G. F., Cleverley, J. S., Hough, R. M., Godel, B., Barnes, S. J., James, S. A., Spiers, K. M., Alfeld, M., Wellenreuther, G., Vukmanovic, Z. & Borg, S. (2019). *J. Phys. Conf. Ser.* **499**, 012002.

[bb27] Schlosser, D. M., Lechner, P., Lutz, G., Niculae, A., Soltau, H., Strüder, L., Eckhardt, R., Hermenau, K., Schaller, G., Schopper, F., Jaritschin, O., Liebel, A., Simsek, A., Fiorini, C. & Longoni, A. (2010). *Nucl. Instrum. Methods Phys. Res. A*, **624**, 270–276.

[bb28] Schneider, G., Niemann, B., Guttmann, P., Rudolph, D. & Schmahl, G. (1995). *Synchrotron Radiat. News*, **8**(3), 19–28.

[bb29] Schroer, C. G., Boye, P., Feldkamp, J. M., Patommel, J., Samberg, D., Schropp, A., Schwab, A., Stephan, S., Falkenberg, G., Wellenreuther, G. & Reimers, N. (2010). *Nucl. Instrum. Methods Phys. Res. A*, **616**, 93–97.

[bb22] Seiboth, F., Schropp, A., Scholz, M., Wittwer, F., Rödel, C., Wünsche, M., Ullsperger, T., Nolte, S., Rahomäki, J., Parfeniukas, K., Giakoumidis, S., Vogt, U., Wagner, U., Rau, C., Boesenberg, U., Garrevoet, J., Falkenberg, G., Galtier, E. C., Ja Lee, H., Nagler, B. & Schroer, C. G. (2017). *Nat. Commun.* **8**, 14623. 10.1038/ncomms14623 PMC5337966 28248317

[bb30] Senkbeil, T., Mohamed, T., Simon, R., Batchelor, D., Di Fino, A., Aldred, N., Clare, A. S. & Rosenhahn, A. (2016). *Anal. Bioanal. Chem.* **408**, 1487–1496. 10.1007/s00216-015-9253-6 26715248

[bb31] Solé, V. A., Papillon, E., Cotte, M., Walter, P. & Susini, J. (2007). *At. Spectrosc.* **62**, 63–68.

[bb32] Stuhr, S., Truong, V. K., Vongsvivut, J., Senkbeil, T., Yang, Y., Al Kobaisi, M., Baulin, V. A., Werner, M., Rubanov, S., Tobin, M. J., Cloetens, P., Rosenhahn, A., Lamb, R. N., Luque, P., Marchant, R. & Ivanova, E. P. (2018). *Sci. Rep.* **8**, 8413. 10.1038/s41598-018-26563-6 PMC5976759 29849036

[bb33] Takeuchi, A., Terada, Y., Suzuki, Y., Uesugi, K. & Aoki, S. (2009). *J. Synchrotron Rad.* **16**, 616–621. 10.1107/S0909049509029598 19713634

[bb3] Todd, A. C. & Chettle, D. R. (1994). *Res. Adv.* **102**, 172–177. 10.1289/ehp.94102172 PMC1567203 8033846

[bb34] West, M., Ellis, A. T., Potts, P. J., Streli, C., Vanhoof, C., Wegrzynek, D. & Wobrauschek, P. (2012). *J. Anal. At. Spectrom.* **27**, 1603–1644.

[bb35] Wu, Y., Cai, B., Feng, W., Yang, B., Huang, Z., Zuo, C. & Wang, L. (2014). *Ind. J. Med. Res.* **140**, 513–519. PMC4277137 25488445

