# Supplementary material for: X-ray fluorescence analysis of metal distributions in cryogenic biological samples using large-acceptance-angle SDD detection and continuous scanning at the Hard X-ray Micro/Nano-Probe beamline P06 at PETRA III
Source: J Synchrotron Radiat. 2020 Jan 1;27(Pt 1):60–6. doi: 10.1107/S1600577519014048 (PMC6927521; doi:10.1107/S1600577519014048)
Supplement: Supplementary file 1 [file s-27-00060-sup1.pdf]

JOURNAL OF SYNCHROTRON RADIATION

Volume 27 (2020)

Supporting information for article:

X-ray fluorescence analysis of metal distributions in cryogenic biological samples using large-acceptance-angle SDD detection and continuous scanning at the Hard X-ray Micro/Nano-Probe beamline P06 at PETRA III

C. Rumancev, A. Gräfenstein, T. Vöpel, S. Stuhr, A. R. von Gundlach, T. Senkbeil, J. Garrevoet, L. Jolmes, B. König, G. Falkenberg, S. Ebbinghaus, W. H. Schroeder and A. Rosenhahn

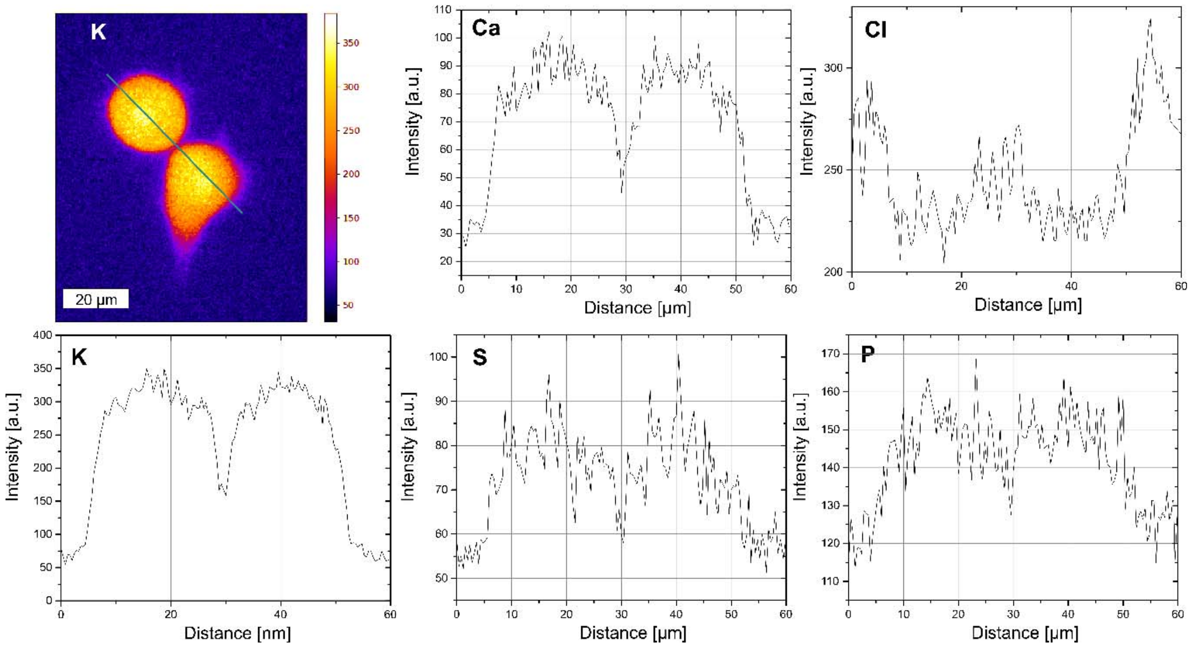

Figure S1 Intensity line profiles across the cells shown in figure 5. The position of the line profile is shown in the potassium map.

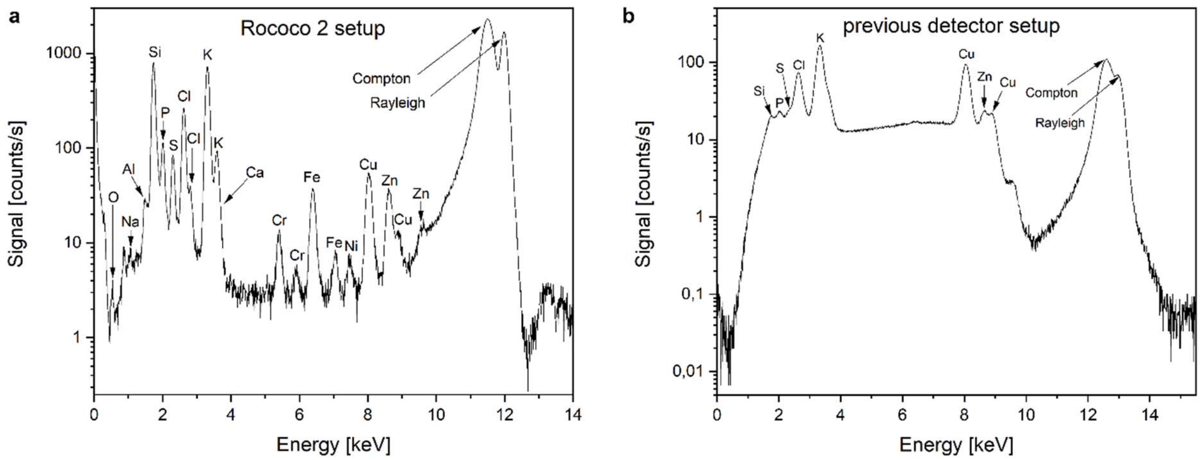

Figure S2 Comparison of XRF sum spectra over 100 pixels obtained on similar single HeLa cells with the new Rococo 2 setup (a) and the previously used setup with a low acceptance angle of 0.066 Sr (b). Exposure time was 0.1 s for the Rococo 2 setup and 1.5 s for the previous setup. The spectra were collected using an X-ray focus size of 300 nm x 300 nm with an excitation energy of 12 keV (left) and 600 nm x 530 nm with an excitation energy of 13 keV (right). For the spectrum of the prior detector setup a much larger background is evident while only a fraction of the elemental peaks in the Rococo 2 spectrum could be resolved.

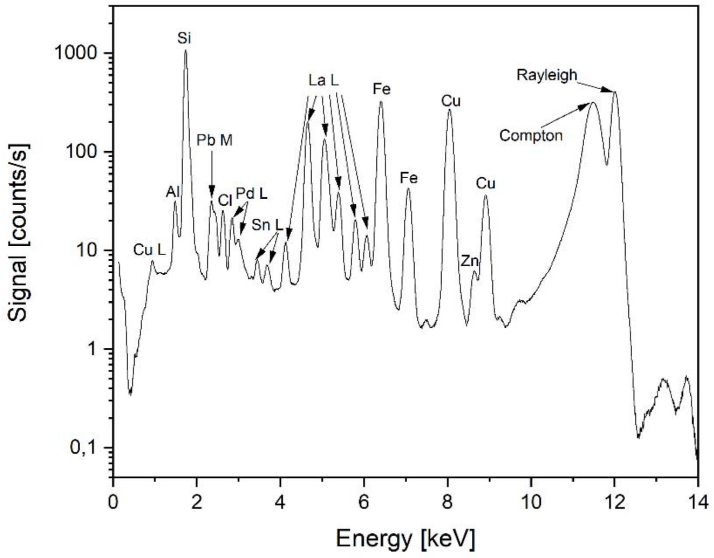

Figure S3 XRF sum spectrum over 100 pixels obtained with the Rococo 2 detector on the thin film XRF reference sample „RF17-200-S4218-41“ (AXO Dresden, Germany). The sample contains thin films of the following elements: Pb, La, Pd, Mo, Cu and Fe. XRF peaks for all the listed elements could be except for Mo. The Mo Lα has an energy of 2.29 keV and thus is in the energy range of the spectrum but cannot be seen due to an overlap with the low-energy tail of the Pb Mα peak. The concentration of Mo was the lowest of the elements with 50 ppm, followed by Pd with 95 ppm. As the L transition peaks of Pd could be clearly resolved, a sample and element depending trace order of magnitude of the detection limit between 50 – 95 ppm can be estimated. The lowest resolved peak is Cu Lα with an energy of 0.93 keV. Exposure time was 10 s per pixel, spot size was 300 nm x 300 nm and excitation energy was 12 keV.
